# Development of a Novel Human CD147 Transgenic NSG Mouse Model to test SARS-CoV-2 Infection and Immune Responses

**DOI:** 10.21203/rs.3.rs-396257/v1

**Published:** 2021-04-07

**Authors:** Saiaditya Badeti, Hsiang-chi Tseng, Peter Romanienko, Ghassan Yehia, Dongfang Liu

**Affiliations:** Rutgers University: Rutgers The State University of New Jersey; Rutgers University: Rutgers The State University of New Jersey; Rutgers Cancer Institute of New Jersey; Rutgers Cancer Institute of New Jersey; Rutgers New Jersey Medical School

**Keywords:** COVID-19, SARS-CoV-2, Spike protein, CD147, transgenic mouse model, NSG, physiological expression

## Abstract

An animal model that can mimic the SARS-CoV-2 infection in humans is critical to understanding the newly emerged, rapidly spreading SARS-CoV-2 and development of therapeutic strategies. Studies show that the spike (S) proteins of SARS-CoV (SARS-CoV-S-1-S) and SARS-CoV-2 (SARS-CoV-2-S) bind to human angiotensin-converting enzyme 2 (hACE2, a well-recognized, functional receptor for SARS-CoV and SARS-CoV-2) to mediate viral entry. Several hACE2 transgenic (hACE2Tg) mouse models are being widely used, which is clearly invaluable. However, the hACE2Tg mouse model cannot fully explain: **1)** low expression of ACE2 observed in human lung and heart, but lung or heart failure occurs frequently in severe COVID-19 patients); **2)** low expression of ACE2 on immune cells, but lymphocytopenia occurs frequently in COVID-19 patients; and **3)** hACE2Tg mice do not develop strong clinical disease following SARS-CoV-2 infection in contrast to SARS-CoV-1. Moreover, one of most outstanding features of coronaviruses is the diversity of receptor usage, which includes the newly proposed human CD147 (hCD147) as a receptor for SARS-CoV-2-S. It is still debatable whether CD147 can serve as a functional receptor for SARS-CoV-2 infection or entry. Here we successfully generated a hCD147Tg mouse model in the NOD-*scid* IL2Rgamma^null^ (NSG) background. In this hCD147Tg-NSG mouse model, the hCD147 genetic sequence was placed following the endogenous mouse promoter for mouse CD147 (mCD147), which creates an in vivo model that may better recapitulate physiological expression of CD147 proteins at the molecular level compared to the existing and well-studied K18-hACE2-B6 model. In addition, the hCD147Tg-NSG mouse model allows further study of SARS-CoV-2 in the immunodeficiency condition which may assist our understanding of this virus in the context of high-risk populations with immunosuppressed conditions. The hCD147Tg-NSG mouse mode can serve as an additional animal model for interrogate whether CD147 serve as an independent functional receptor or accessory receptor for SARS-CoV-2 entry and immune responses.

## Introduction:

SARS-CoV-2 is the novel coronavirus that causes Coronavirus-Disease 2019 (COVID-19) and has become a global pandemic and devastated millions. To date, there have been over 128 million confirmed cases of COVID-19 resulting in over 2.8 million deaths (WHO COVID-19 Dashboard. Geneva: World Health Organization, 2020. Available online: https://covid19.who.int/ (last cited: [04/01/21])). While there are many similarities between SARS-CoV-2 and its sister virus SARS-CoV-1 [1], there are many differences that have been uncovered over the past year [2]. For example, many antibodies derived from convalescent patients who developed a successful immune response against the SARS-CoV-1 virus were unable to demonstrate effective neutralization capacity against SARS-CoV-2 pseudovirus and prevent entry into target cells expressing the angiotensin converting enzyme 2 protein (ACE2) [3]. ACE2 represents the dominant entry receptor for both SARS-CoV-1 and SARS-CoV-2 via engagement with the virion’s spike (S) protein [4]. However, it has been recently shown that due to the presence of various mutations in the RBD domain of SARS-CoV-2, its ability to bind to ACE2 can be dramatically increased thus potentially increasing viral entry [5], escaping of antibody responses [6], and propagation of new variants through populations [7]. It has been also demonstrated that a proteolytic receptor called Transmembrane protease serine 2 (TMPRSS2) also plays a significant role in priming the SARS-CoV-2 spike protein and facilitating membrane fusion [8, 9]. Recently, CD26 has also been propounded as an entry protein [10]. Another receptor called basigin, also known as CD147, has been recently postulated to serve as an additional entry receptor for SARS-CoV-2 [11], although it is still debatable [12].

While the fundamental mechanism by which SARS-CoV-2 interacts with CD147 in humans is debated in the scientific community [12], there is a lot of potential rationales supporting the theory that CD147 could still play a role in the COVID-19 clinical course, even if it is not truly a viral entry receptor, in addition to serving as a functional receptor. For example, compared to ACE2 protein expression, CD147 is expressed in cardiomyocytes and endothelial cells, which may correlate with massive hemodynamic instability and cardiovascular abnormalities during COVID-19 infection [13, 14]. Next, CD147 has been shown to serve as the potential entry receptor for a variety of other viral and non-viral pathogens as well, including rhinovirus [15], measles [16], meningitis [17], HIV-1 [17], and malaria into red blood cells [18]. This is potentially one reason that drugs such as hydroxychloroquine and azithromycin, which decreases the entry of *Plasmodium falciparum* [19–21], and Meplazumab (NCT04586153), a humanized anti-CD147 antibody, may have shown efficacy in small clinical studies and through anecdotal evidence during the early days of the pandemic [22–24]. However, because CD147 is diffuse and implicated in many physiological [25] and immune processes [26, 27], a number of indirect mechanisms not related to viral entry may be able to explain these positive finding and provide support for further study in COVID-19 [28, 29].

To this aim, we generated a humanized CD147 transgenic mouse model in the immunocompromised NOD-*scid* IL2Rgamma^null^ (NSG) background, which lacks a functional immune system. We performed preliminary assays to determine whether the expression of human CD147, the purported additional entry receptor for SARS-CoV-2, could sufficiently and independently predispose NSG mice to clinical manifestation of severe COVID-19 disease. The added benefit of the immunocompromised NSG background will allow for scientists interested in studying individual immune cell classes in isolation during the SARS-CoV-2 clinical course through adoptive transfer of immune cells prior to infection. Further studies using this mouse model would be able to determine by what mechanism CD147 increases or decreases viral presence in various organs without the confounding presence of a competent host immune system.

## Results:

### Generation of human CD147 transgenic mouse using CRISPR/Cas9 vector.

**Figure 1:**
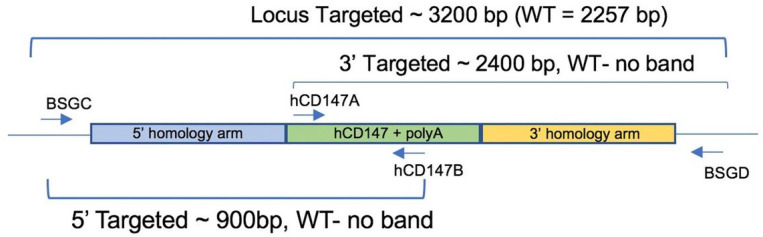
Schematic representation of genotyping primers used to confirm hCD147Tg expression. A combination of 4 primers were used to screen mice (internal primers hCD147A and hCD147B) and confirm proper integration into the mouse CD147 allele (flanking primers BSGC and BSGD).

**Figure 2:**
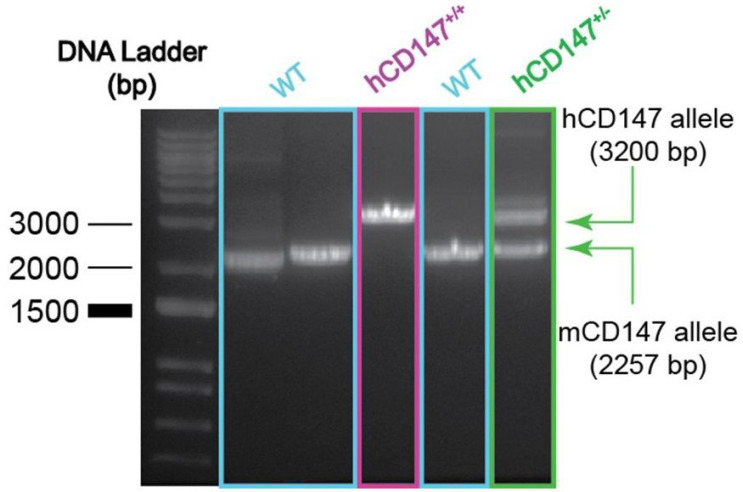
Genotyping of hCD147Tg mouse model. Band patterns emerging from full-length PCR amplification (using BSGC and BSGD primers) of CD147 insertion site. Wild-type (mCD147; 2257 bp) bsg allele is distinguished from the hCD147Tg allele (hCD147; ~3200 bp) in WT-NSG and hCD147Tg-NSG founder mouse (hCD147+/+) and heterozygous hCD147Tg-NSG offspring (hCD147+/−). Standard PCR products were imaged on a gel documentation system.

We developed a mouse model in which hCD147 was expressed into mice whose normal cells and tissue express a hCD147 transgene at hemizygous levels and homozygous levels (Fig. 1). Specifically, a human cDNA encoding CD147 was targeted to mouse CD147 exon 1. The resulting knock-in created a fusion protein with the 1st 22 amino acids of mouse CD147 signal peptide and amino acids 23–385 of human CD147 (NP_001719.2) that is expressed from the mouse CD147 promoter. Transcription termination was mediated by a bovine growth hormone polyadenylation signal sequence. Targeting was performed directly in NSG mouse embryos (JAX stock#: 005557) by co-injecting a targeting vector and Cas9 protein complexed with a CRISPR sgRNA recognizing and cutting the sequence 5’-GCCTGCGCGGCGGGTAAGAG-3’. Fourteen positive founders were determined to be correctly targeted by PCR genotyping and subsequent sequencing of the targeted alleles in their entirety. Three of the 14 were determined to be biallelic at the locus. The hCD147 frequencies and antigen density are close to human CD147 expression patterns in humans. After completing the construct, we generated the mice and performed phenotype verification. Correct genotyping of hCD147Tg mice was observed (Fig. 2). A representative genotype confirms the successful generation of the hCD147Tg mouse (Fig. 2), and the genotyping products were verified by DNA sequencing (data not shown).

### Verification of human CD147 protein expression in various tissues by immunohistochemistry.

**Figure 3:**
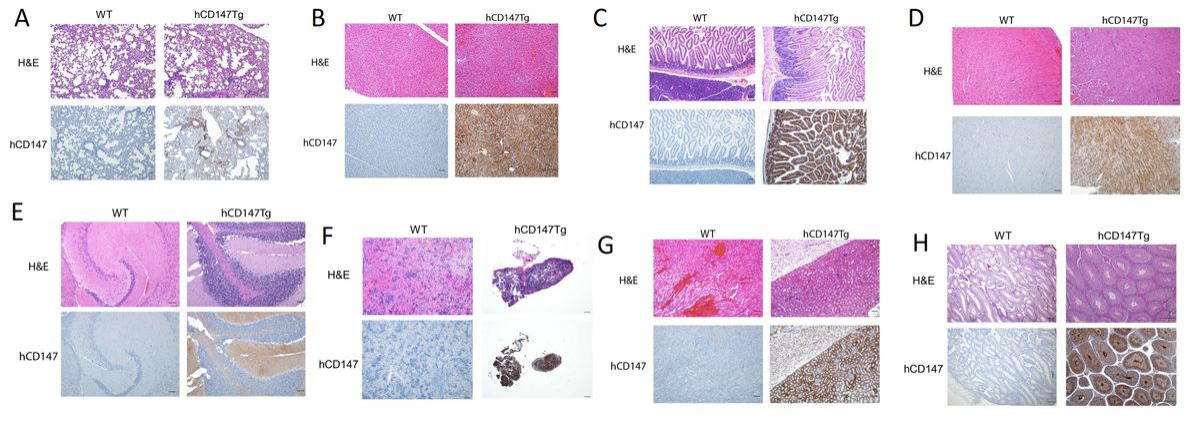
H&E and immunohistochemistry of human CD147 in hCD147Tg-NSG mice. Human CD147 was stained (HIM6; 1:500) in the lung (3A), liver (3B), intestine (3C), heart (3D), brain (3E), spleen (3F), kidney (3G), testes (3H) in WT-NSG (top) and hCD147Tg-NSG (bottom) mice. Images were taken using an Olympus Inverted Light Microscope. Scale bar represents 200 μm.

After successful generation of hCD147Tg-NSG mice, we further verified human CD147 protein expression in these mice. Organs were harvested from adult hCD147Tg-NSG mice and WT-NSG littermates and stained for human CD147 protein by immunohistochemistry (Fig. 3). We observed strong and specific hCD147 protein staining across all tissues assayed (lung, **3A** liver, **3B**; intestine, **3C**; heart, **3D**; brain, **3E**; spleen, **3F**; kidney, **3G**; testes, **3H**) in transgenic mice compared to WT-NSG mice where no staining was visualized. Interestingly, we observed that red blood cells in the lung also expressed strong human CD147 staining indicating successful integration of the protein on erythrocyte precursors and penetration into bone marrow. The strongest staining was associated with bronchioles in the lungs, in condensing spermatids in the testes, and in mucosal villi in the intestines

### Verification of co-expression of both hCD147 and mCD147 in various tissues and blood in hCD147Tg-NSG.

**Figure 4:**
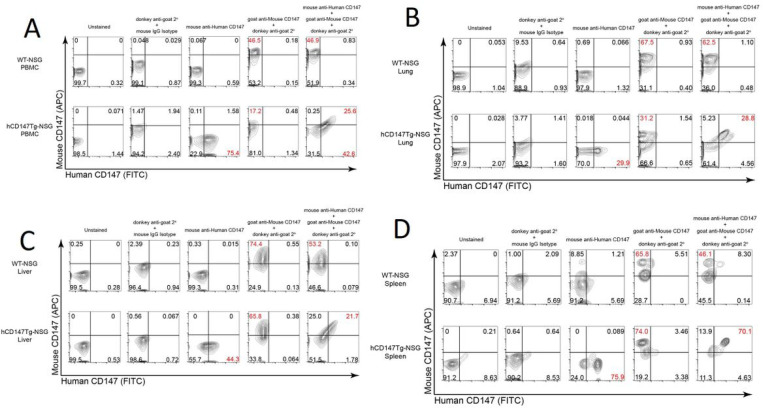
Flow cytometric analysis reveals proper dual-expression of both mCD147 and hCD147 in PBMC and various organs. Representative contour plots of CD147 expression on WT-NSG (top) and hCD147Tg-NSG (bottom) cells from PBMC (4A), lung (4B), liver (4C), spleen (4D) using antibodies targeting either mouse CD147 protein, human CD147 protein, or a combination of both antibodies (far right). Relative percentages are listed, and significant shifts highlighted in red. Gating was determined based on donkey anti-goat / mouse isotype IgG antibody background staining.

To confirm proper expression both wild-type mouse and transgenic human CD147 in hCD147Tg-NSG mice, we first validated that our antibodies would not show inter-species cross-reactions by testing them against the human cell line HepG2 and the mouse cell line BNL 1 ME A.7R.1 (Figure S1). Observing no sign of inter-species cross-reaction, we then analyzed the expression of both proteins in various organs of hCD147Tg-NSG and WT-NSG mice by flow cytometry (Fig. 4). Successful co-expression of both receptors in hCD147Tg-NSG mice was observed in the peripheral blood mononuclear cells (**4A**), spleens (**4B**), lungs (**4C**), and livers (**4D**) of transgenic mice but not in WT-NSG littermates. Together, we successfully generated the hCD147Tg-NSG mouse model, which can be used to test SARS-CoV-2 infection in vivo. This mouse will be available for scientific communities.

## Materials And Methods:

### Genotyping for hCD147Tg-NSG mice:

DNA from ear snips or toes was extracted using the HOTSHOT method for DNA preparation. Briefly, mice tissues were placed in 50μl of alkaline lysis solution (25mM NaOH, 0.2mM EDTA) and heated at 94°C for 30–60 minutes. 50μl of neutralization solution (40mM Tris-HCl, pH 5.0) was then added to preserve DNA. To screen mice for the hCD147 wild-type cDNA fragment, the following primers were used to generate a 223-base pair (bp) PCR fragment: 5’-GAAAACGGAGTTCAAGGTGGACTC-3’ (hCD147A) and 5’-TCAGAGTCAGTGATCTTGTACCAG-3’ (hCD147B). To confirm proper CRISPR integration of the entire hCD147 construct within the mouse *bsg* allele DNA from toe or tail biopsies were extracted using Qiagen QIAmp DNA mini kit (Qiagen, cat#51306) and the following primers were used to generate to ~ 3200 bp hCD147 transgenic fragment that can be distinguished from the ~ 2257 bp WT fragment: 5’-GAAAAGGACAGCCGAGCATCGTG-3’ (BSGC) and 5’-TGTGGACTATGGAGAACCTGCAAG-3’ (BSGD). To confirm the 5’ arm, a combination of BSGC and hCD147B primers were used to generate a ~ 900 bp fragment. To confirm the 3’ arm, a combination of hCD147A and BSGD primers were used to generate a ~ 2400 bp fragment.

### Immunohistochemistry for CD147 on FFPE slides.

Immunohistochemistry was performed on FFPE tissue slides following a 45-minute antigen retrieval step (pH 6). To stain human CD147 (hCD147), the Dako EnVision + System- HRP Labelled Polymer kit (Agilent) was used according to manufacturer’s instructions against slides incubated with primary mouse anti-human CD147 antibody (Biolegend, HIM6; 1:500). To stain mouse CD147 (mCD147), polyclonal donkey anti-goat IgG secondary (Jackson ImmunoResearch, 705–035-003 [HRP]; 1:500) was used against primary goat anti-mouse CD147 antibody (R&D Systems, BAF772; 1:100). Slides were then incubated with 3,3′-Diaminobenzidine (DAB) for 4 minutes before washing and counterstained with Gill No. 2 hematoxylin. Slides were then dehydrated and mounted.

### Single-cell isolation of organ tissue and flow cytometry staining:

Organs and peripheral blood were obtained from euthanized adult hCD147Tg-NSG and WT-NSG mice. Organs were then mechanically and chemically digested with Collagenase IV (Gibco, 17104019) for 10 minutes using the gentleMACS Octo with heater (Miltenyi) before triturating through a 70μM cell strainer. The strained fraction was centrifuged at 400g for 5 minutes and then resuspended in ACK Lysis Buffer (Gibco, A1049201) for 5 minutes on ice. Phosphate-buffered saline (PBS) was added to quench the reaction and the cell suspension was centrifuged again. The supernatant was discarded, and the cell pellet was divided into various sample groups. Mouse cells were first preincubated with Fc Block according to manufacturer’s recommendations before proceeding to antibody incubation. Mouse CD147 was stained using primary goat anti-mouse CD147 (R&D Systems, BAF772) and visualized using Cy5-conjugated polyclonal donkey anti-goat IgG secondary (Jackson ImmunoResearch, 705–175-147). Human CD147 was stained using primary FITC-conjugated mouse anti-human CD147 antibody (Invitrogen, MEM-M6/1) Antibodies were applied at a 1:100 dilution per sample for 30 minutes on ice, rinsed with PBS, and resuspended in PBS. Acquisition was performed on a BD Accuri™ C6 Plus system and downstream analysis was done using FlowJo (Treestar).

## Discussion:

Key to the development of successful and effective vaccines to SARS-CoV-2 infections and treatments for COVID-19 patients is to understand SARS-CoV-2 infectivity and pathogenesis. The fundamental mechanism underlying the SARS-CoV-2 entry remains poorly understood. Previous studies show that the spike (S) proteins of SARS-CoV-1 (SARS-CoV-S [4, 30]) and SARS-CoV-2 (SARS-CoV-2-S [31–35]) bind to human angiotensin-converting enzyme 2 (hACE2), a well-recognized, functional receptor for SARS-CoV-1 and SARS-CoV-2, to mediate viral entry. A hACE2 transgenic (Tg) mouse model is being widely used [36–39], which is clearly invaluable but with some limits (e.g., low expression of hACE2 in human lung, heart, and immune cells). Other models for studying SARS-CoV-2 infection in mice that are currently being optimized include models that utilize mouse-adapted virus derivations [40, 41], immunocompromised or obese mice that lack interferon receptors [41], or utilize different animals entirely [42, 43]. However, while these models have recapitulated some aspects of the COVID-19 disease course in infected mice, such as lung inflammation [44], cytokine storm [45], viral neuroinvasion [46], and impaired lung function [47], they fail to explain other aspects of COVID-19 such as increased thrombosis risk for affected individuals, increased risk for COVID-19 in diabetic patients [48], hemodynamic instability, and why stroke and immunosuppression may be predisposing risk factors and clinical sequelae of COVID-19 [49–51].

One of most outstanding features of coronaviruses is the diversity of receptor usage, which includes the newly proposed human CD147 (hCD147) as a receptor for SARS-CoV-2. CD147 is a transmembrane glycoprotein with multiple functions in normal lung, immune cells, and diseased tissues [52]. The role of CD147 in normal lung remains obscure. The role of CD147 in immune cells is important for T cell activation and proliferation, as well as cell migration, adhesion, and invasion [52]. CD147 is expressed on different cell types (e.g., hematopoietic, epithelial, and endothelial cells) at varying levels [53]. Normal epithelial and fetal tissues have low expression of CD147, when measured by immunohistochemical analysis [54]. However, CD147 is significantly upregulated in aggressive and chronic disease states, such as in cancers [55, 56], atherosclerosis [57], diabetes [58], ischemic stroke [59], and chronic lung obstruction diseases [60]. Intriguingly, recent studies show that CD147 plays a functional role in facilitating SARS-CoV-1 and SARS-CoV-2 entry [22, 61], and antibody against CD147 blocks the infection of SARS-CoV-2 [22]. A humanized anti-CD147 antibody (Meplazumab) efficiently improves the recovery of COVID-19 patients with pneumonia with a favorable safety profile [62]. However, the majority of studies related to CD147 and SARS-CoV-2 are focused on cell line-based *in vitro* assays and protein binding experiments and have yet-to-be verified *in vivo* [63, 64].

Our hCD147Tg model offers several strengths to the scientific community as it will better capture other nuances of the COVID-19 disease. **(1)** This model will allow researchers to study hemodynamic instability and increased thrombosis risk following COVID-19 infection as the hCD147 protein is expressed in circulating erythrocytes. **(2)** The NSG background will allow scientists to study how adoptive transfer of any immune cell subset can either dampen or initiate the COVID-19-induced cytokine storm that is often seen in affected individuals. Additionally, as the NSG background has been used to study diabetes [65], our model will allow further studies into the role of diabetes and COVID-19. **(3)** This model can be crossed with other mouse models to determine whether a combination of human CD147 and other viral entry-related receptors (e.g., ACE2, TMPRSS2, CD26) can exacerbate clinical disease. **(4)** As the human CD147 protein is expressed at physiological levels in these mice, this model will better recapitulate true physiological conditions and expression patterns normally observed in mice and humans. Even if CD147 is later determined to play a relatively minor role compared to ACE2 in SARS-CoV-2 viral entry, this mouse model may prove to be invaluable for understanding how the virus globally impacts CD147-positive cells and tissues in the in vivo setting and how therapies may modulate COVID-19 disease via this receptor. **(5)** This model can be used to test the infectivity and pathogenesis of the emergence of variants of SARS-CoV-2, such as B.1.1.7, B.1.351, and P1 (20J501Y.V3), given the recently reported studies showing extension of host range to BALB/c and C57BL/6 mice [66]. In conclusion, the newly generated hCD147Tg-NSG mouse model can be used as a platform where direct clinical implications for vaccine and therapeutic strategies can be evaluated.

## Supplementary Material

Supplement

Supplement
