# Supplementary material for: Development of a Novel Human CD147 Transgenic NSG Mouse Model to test SARS-CoV-2 Infection and Immune Responses
Source: Res Sq. 2021 Apr 7:rs.3.rs-396257. Preprint. [Version 1] doi: 10.21203/rs.3.rs-396257/v1 (PMC8043462; doi:10.21203/rs.3.rs-396257/v1)
Supplement: Supplement [file 12fd6cb119ba1388e9d45558.docx]

**Supplementary Figure legend:**

**Figure S1**: **Species specificity of anti-human CD147 and anti-mouse CD147 antibodies tested against human and mouse cell lines**. Representative contour plots of CD147 expression on BNL 1ME A.7R.1 (top) and HepG2 (bottom) cells using antibodies targeting either mouse CD147 protein, human CD147 protein, or a combination of both antibodies (far right). Relative percentages are listed, and significant shifts highlighted in red. Gating was determined based on donkey anti-goat and mouse isotype IgG antibody background staining.
