# Supplementary figures and images for: Development of a Novel Human CD147 Transgenic NSG Mouse Model to test SARS-CoV-2 Infection and Immune Responses
Source: Res Sq. 2021 Apr 7:rs.3.rs-396257. Preprint. [Version 1] doi: 10.21203/rs.3.rs-396257/v1 (PMC8043462; doi:10.21203/rs.3.rs-396257/v1)

## Slide 1
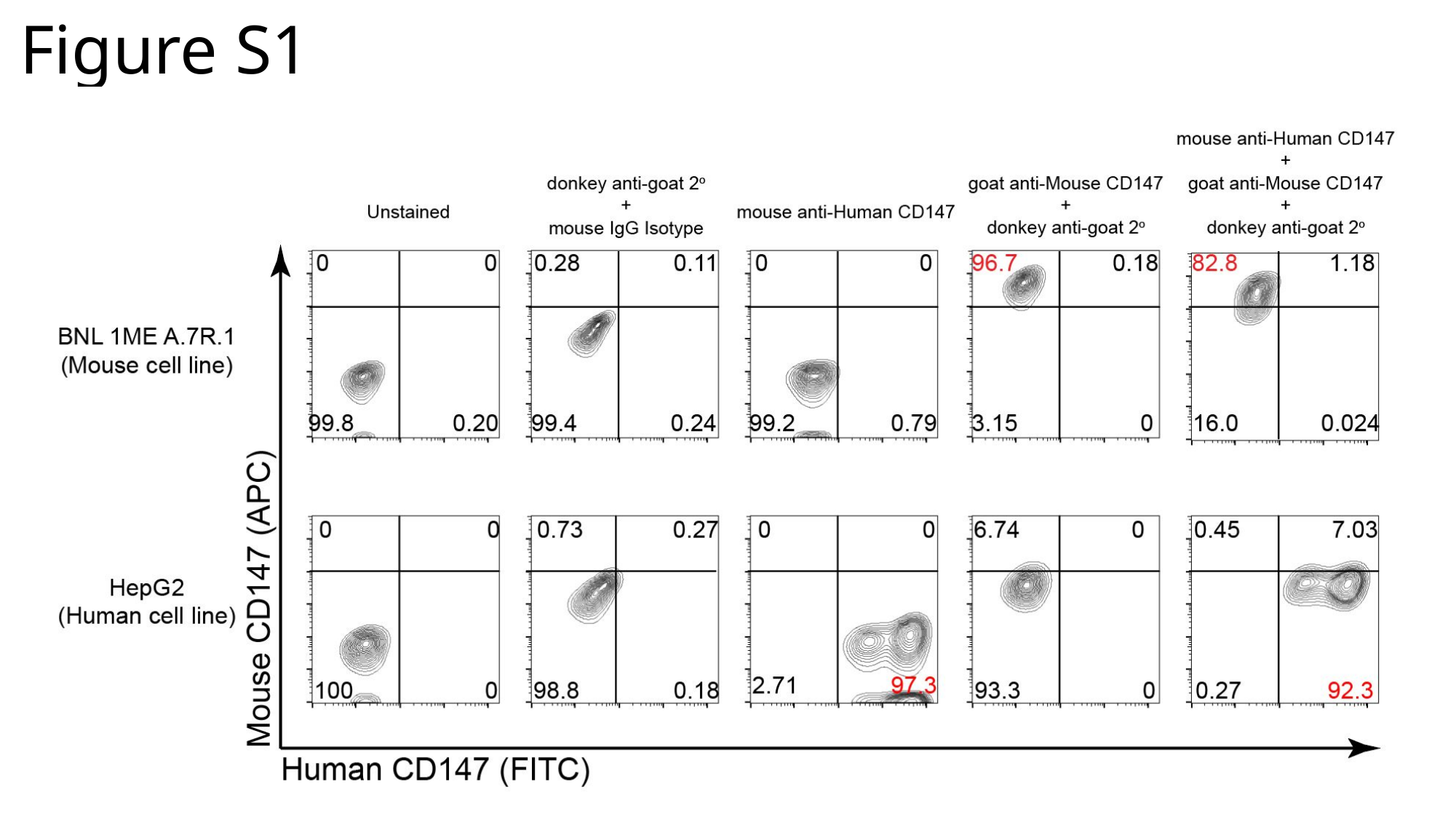

# Figure S1

Supplement: Supplement [file c4effc4c3868a92e09fb5a2e.pptx]
